# Outcomes of remnant‐preserving anterior cruciate ligament reconstruction using Achilles tendon allografts: A retrospective analysis of tibial tunnel widening and complications

**DOI:** 10.1002/jeo2.70231

**Published:** 2025-04-04

**Authors:** Siyeon Kim, Dae Keun Suh, Dong Won Suh

**Affiliations:** ^1^ Department of Orthopedic Surgery Samsung Medical Center, Sungkyunkwan University School of Medicine Seoul Republic of Korea; ^2^ Department of Clinical Research Design and Evaluation SAIHST, Sungkyunkwan University Seoul Republic of Korea; ^3^ Department of Orthopaedic Surgery Kyung Hee University Hospital Seoul Republic of Korea; ^4^ Joint Center Barunsesang Hospital Seongnam‐si Gyeonggi‐do Republic of Korea

**Keywords:** Achilles tendon, allograft, anterior cruciate ligament, reconstruction, tibial tunnel widening, transtibial

## Abstract

**Purpose:**

This study evaluates the outcomes of remnant‐preserving Anterior cruciate ligament reconstruction (ACLR) using Achilles tendon allografts, focusing on tibial tunnel widening and complications. The primary research question is whether this technique results in significant tunnel widening and other complications.

**Methods:**

We performed a retrospective analysis of 396 patients who underwent ACLR with Achilles tendon allografts between 2010 and 2023. Magnetic resonance imaging (MRI) scans were taken post‐operatively and at follow‐up, with tunnel width measured at both time points. Tunnel width was measured perpendicular to the tunnel's long axis, assessing both anteroposterior and mediolateral dimensions, 1 cm distal to the articular surface. The cross‐sectional area (CSA) of the tibial tunnel was calculated by multiplying the two measured widths. Statistical analysis included paired *t* tests and linear regression to assess factors affecting tibial tunnel widening.

**Results:**

Follow‐up MRI scans of 179 patients showed a non‐significant increase in the tibial tunnel CSA of 3.49 mm^2^ (*p* = 0.08). However, 45.2% of patients had a decrease in CSA. A statistically significant correlation was found between the time interval between MRI scans and CSA changes, with a decrease in CSA of 0.195 mm^2^ per month (*p* = 0.007). Graft failure occurred in 5.3% of patients, and 2% required additional surgery for meniscal or cartilage damage. Cyclops lesions were identified in six patients and were treated surgically.

**Conclusion:**

This study demonstrates that remnant‐preserving ACL reconstruction using Achilles tendon allografts does not lead to significant tibial tunnel widening and has low complication rates, indicating that it is a safe and effective technique.

**Level of Evidence:**

Level IV, case series.

**Abbreviations**
ACL: anterior cruciate ligament
ACLR: anterior cruciate ligament reconstruction
BMI: body mass index
BTB: bone‐patellar tendon‐bone
CSA: cross‐sectional area
MRI: magnetic resonance imaging
POD: post‐operative day

## INTRODUCTION

The rising popularity of physical activity among young individuals has led to a surge in anterior cruciate ligament (ACL) tears, making this one of the most frequently reported sports‐related injuries [12]. Notably, professional athletes face an annual ACL tear incidence of nearly 3% [17]. Furthermore, patients who experience ACL tears are at increased risk of developing post‐traumatic osteoarthritis, underscoring the importance of effective management strategies to ensure optimal clinical outcomes [25]. Consequently, ACL reconstruction (ACLR) surgeries are becoming increasingly common. However, complications such as tunnel widening and graft re‐tear remain significant challenges following these procedures [5, 10, 26, 30].

Tunnel widening of the femoral and tibial tunnels is a common complication following ACLR, which has been reported to cause graft laxity and influence re‐rupture [31]. Tunnel widening occurs due to biological factors, such as synovial fluid and mechanical factors, including graft motion, improper graft position, graft tension and excessive rehabilitation [26]. Previous studies have attempted to prevent tunnel widening by exploring factors such as graft type, tunnel positioning and screw fixation methods. However, most of these studies have used autografts or had small sample sizes [1, 2, 6, 10, 15, 27].

Hamstring autografts, though commonly used, may inadequately control tibial rotation, contributing to rotational instability. Harvesting gracilis and semitendinosus tendons in cases involving ACL and medial collateral ligament injury can exacerbate valgus rotation [8, 13, 19]. Comparatively, grafts such as quadriceps tendons or iliotibial bands have demonstrated superior control of internal rotation and pivot shifts [7]. In patients with hamstring autografts, the infection rate has been reported to be as high as 1% [32]. It is known that low‐grade infections can lead to graft failure in approximately 20% of cases with primary ACL grafts [3].

In this study, to address the drawbacks associated with autografts, a remnant‐preserving technique using Achilles allograft was applied. By analyzing tibial tunnel widening using MRI scans obtained immediately post‐surgery and during follow‐ups, the study aims to investigate the effect of Achilles tendon allograft on tibial tunnel widening.

## METHODS

### Patients

This study included patients who underwent ACLR performed by a single surgeon at a single institution between 2010 and 31 December 2023. Inclusion criteria were patients aged 18–40 years at the time of surgery. Patients over 40 were excluded due to potential confounding factors from ACL degeneration [21, 28], so only younger patients were included. To focus on primary ACLR cases, patients who had previously undergone ACLR elsewhere and then had revision surgery at our institution were excluded. Additionally, patients who had undergone osteotomy were also excluded.

At this clinic, routine magnetic resonance imaging (MRI) scans are performed on post‐operative day (POD) 1–2 for a post‐surgery check‐up. MRI scans were also taken during outpatient follow‐ups if patients reported discomfort or before screw removal as part of a routine check‐up. Patients who could not undergo MRI scans during this period due to cost or claustrophobia were excluded. Furthermore, since the study aimed to observe tunnel widening progression, patients who only had an MRI follow‐up once after surgery, within 6 months, were excluded from the study.

The institutional review board approved this study and waived the requirement for informed consent because we used de‐identified data routinely collected during clinic visits.

### Variables

Data were collected from patient medical records, including trauma date, surgery date, surgery location, body mass index (BMI) at admission and MRI dates. In cases where the trauma date was imprecisely documented (e.g., described as ‘2 years ago’ or ‘3 months ago’), the trauma date was estimated retrospectively based on the indicated period from the surgery date.

### Surgical technique and rehabilitation

**Figure 1:**
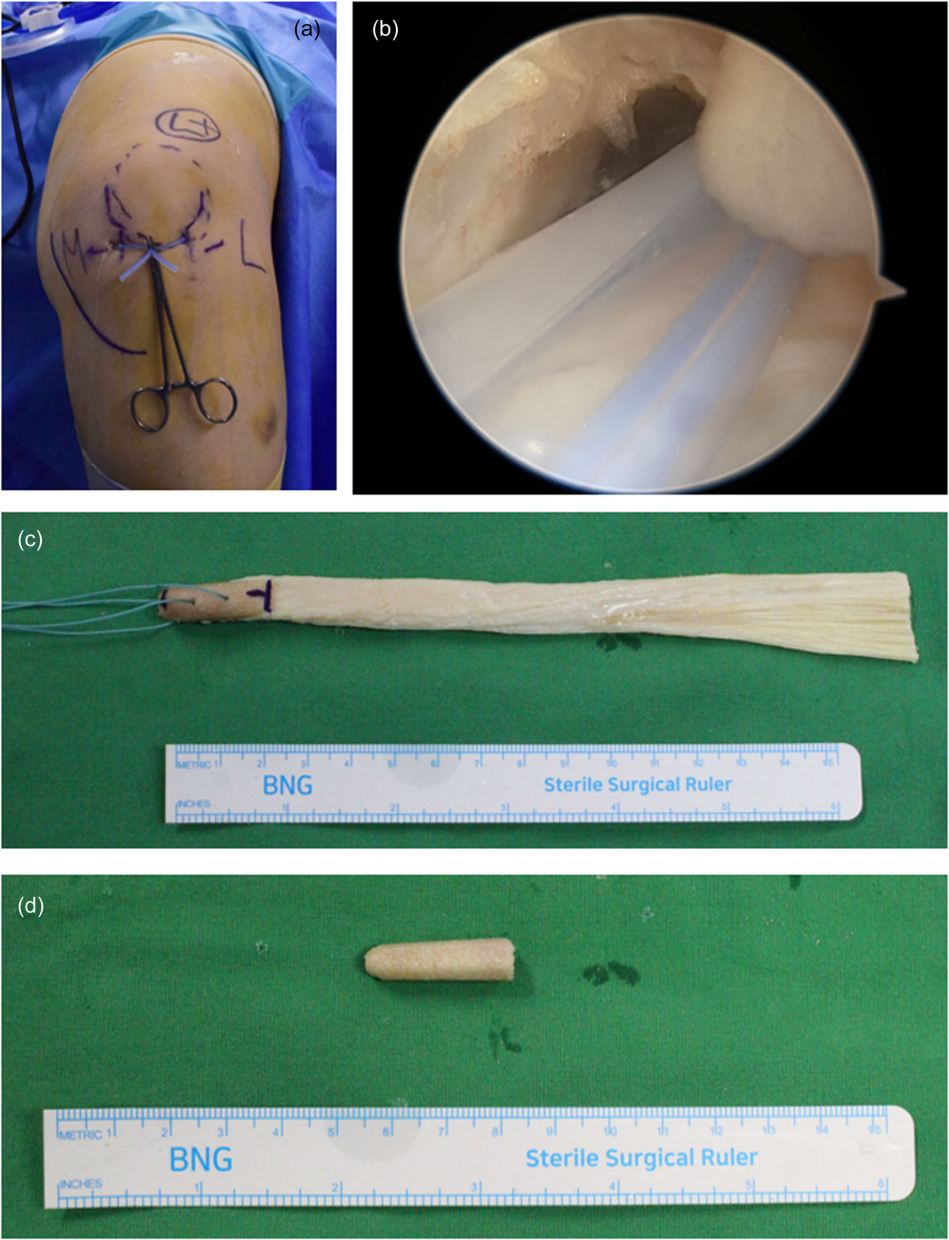
Preparation for surgery. (a) A closed suction drain was inserted between the anteromedial and anterolateral portals to protect the infrapatellar fat pad. (b) An arthroscopic image showing the closed suction drain protecting the infrapatellar fat pad. (c) Prepared allo‐achilles tendon. (d) The calcaneal portion of the allo‐achilles tendon being trimmed to create the tibial tunnel bone plug.

**Figure 2:**
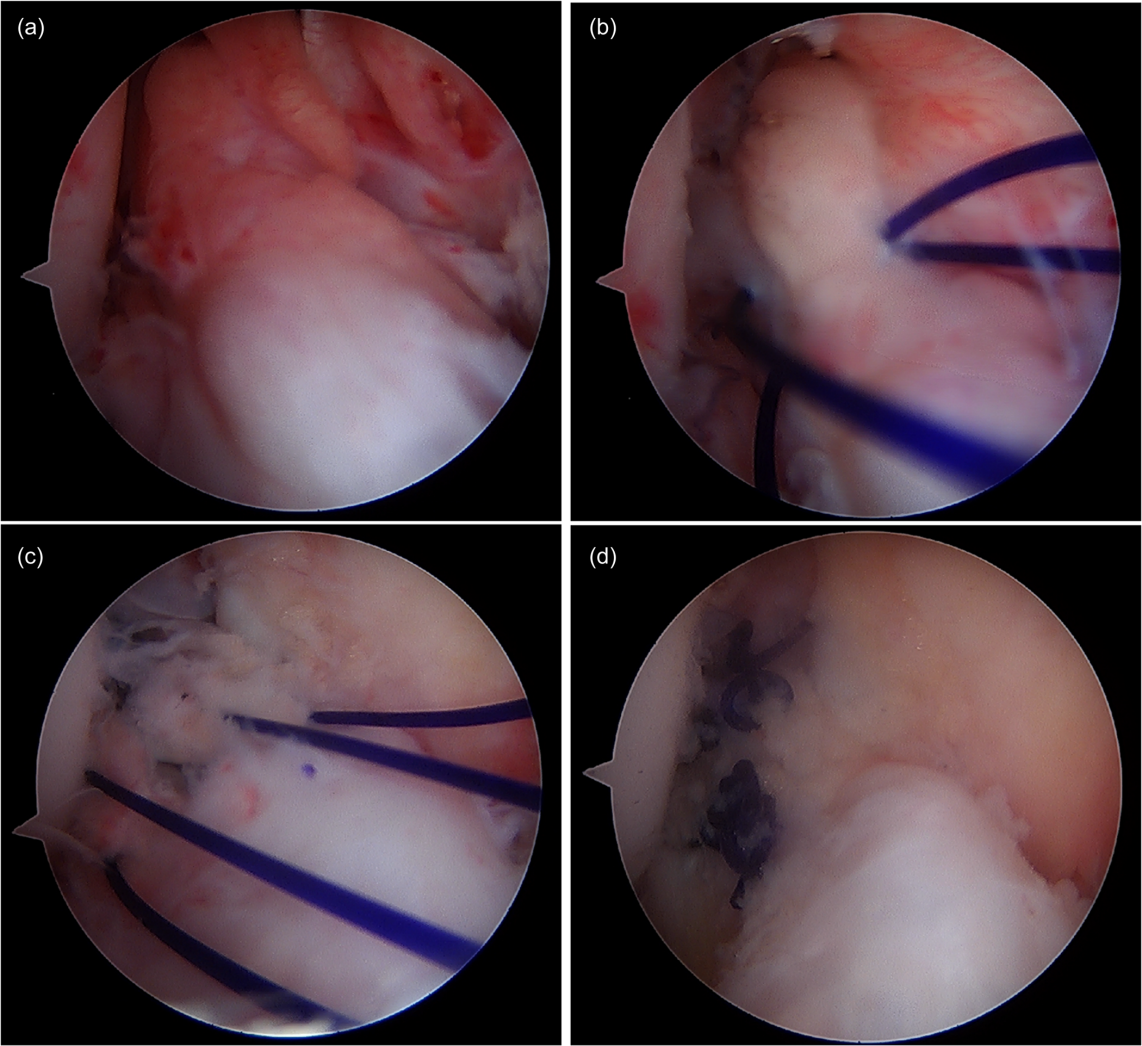
Surgical technique. (a) Remnant ACL tissue, (b) Remnant ACL tissue preserved with PDS, (c) Achilles allograft tendon passing through the tunnel and overlapping the remnant tissue and (d) Allograft and remnant tissue tied to complete the procedure. ACL, anterior cruciate ligament; PDS, polydioxanone synthetic.

The surgical technique followed previously published methods, summarized here for reference [23]. Remnant ACL tissue was preserved using polydioxanone synthetic and Nexon sutures passed through the tissue. A closed suction drain was inserted between the anteromedial and anterolateral portals to protect the infrapatellar fat pad, with removal post‐surgery (Figure 1a,b). A fresh frozen allo‐Achilles tendon, prepared to a thickness of 10 mm and calcaneus portion was trimmed to create a triangular tibial bone plug (Figure 1c,d). Using the transtibial technique, a femoral tunnel was drilled. The prepared allo‐Achilles tendon graft was passed through the reamed tunnel and secured on the femoral side with a metal interference screw. The graft was then pulled out intra‐articularly through the anteromedial portal and fixed at the tibial tunnel using a spiked washer and screw in combination with the tibial bone plug. The remnant ACL tissue and graft were tied together with the previously placed sutures (Figure 2). Post‐operatively, a suction drain was placed in the knee joint, and a cylinder splint was applied. On POD 2, the drain and splint were removed, and an ACL hinge brace was applied for two months. During this time, passive range of motion (ROM) rehabilitation exercises were initiated, and tolerable weight‐bearing with crutch ambulation was permitted from 2 weeks post‐surgery.

### Tibia tunnel width measurements

As mentioned earlier, the patients underwent a routine MRI on POD 1‐2, followed by additional MRIs during outpatient follow‐ups as needed. Knee MRI scans were conducted using 1.5‐T magnets (Siemens and General Electric Health Care) equipped with HD knee coils. The images were captured with moderate echo time settings (repetition time ranging from 2000 to 3800 ms; echo time between 26 and 103 ms). The field of view was 16 cm both in the coronal and sagittal planes, with a matrix size of 320 × 256. Slice thickness was 3 mm in both the coronal and sagittal planes, with a gap of 4.55 mm between slices. Receiver bandwidth was 150–170 Hz/Px.

**Figure 3:**
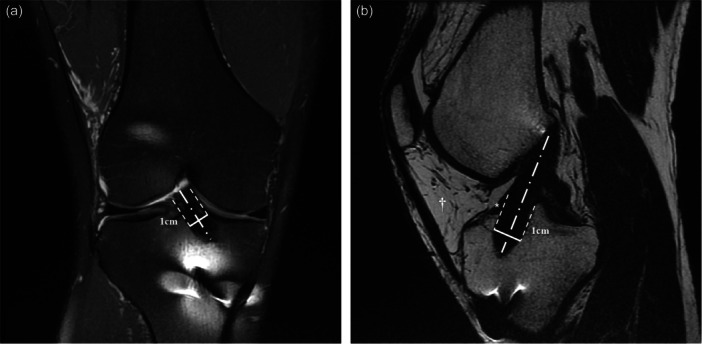
Tunnel width measurements in knee MRI. (a) Coronal view and (b) Sagittal view of the knee, showing tunnel width measured perpendicular to the tunnel's long axis, 1 cm distal to the articular surface. The tunnel's long axis is indicated by the dashed line (alternating long and short dashes), with the perpendicular tunnel width marked by the solid line. In (b), * indicates ACL remnant tissue, and † denotes the preserved fat pad. ACL, anterior cruciate ligament; MRI, magnetic resonance imaging.

Tunnel width measurements followed methods established in previous studies [29]. The PD coronal fat‐sat and T2 oblique sagittal views were selected to ensure alignment with the longitudinal axis of the ACL graft. All tunnel width measurements were performed by a single physician certified in orthopaedic surgery. Tunnel width was measured perpendicularly to the tunnel's long axis, assessing both anteroposterior and mediolateral dimensions 1 cm distal to the articular surface (Figure 3). The cross‐sectional area (CSA) of the tibial tunnel was calculated by multiplying the two measured widths. It was measured twice by a single orthopaedic doctor, and the average value was used for the final calculation. Intra‐rater reliability was assessed by an additional author, and the reliability coefficient was found to be 0.9 with a *p* value of 0.03. As screw fixation was used on the femur, measurements of femoral tunnel widening were not feasible; thus, only the tibial tunnel was analyzed.

### Complications

Complications were evaluated using follow‐up MRI scans and patient medical records. These included graft failure, newly developed meniscal and cartilage damage, infection and the presence of cyclops lesions.

### Statistical analysis

Analysis was conducted using R Studio version 4.4.1, employing Paired *t* tests and linear regression. In the linear regression analysis, the difference in CSA between the initial and follow‐up MRI scans was set as the outcome, adjusting for variables including gender, age, BMI, the duration between trauma and surgery, and the duration between MRI scans. A two‐sided *p* value of less than 0.05 was considered to indicate statistical significance.

## RESULTS

**Figure 4:**
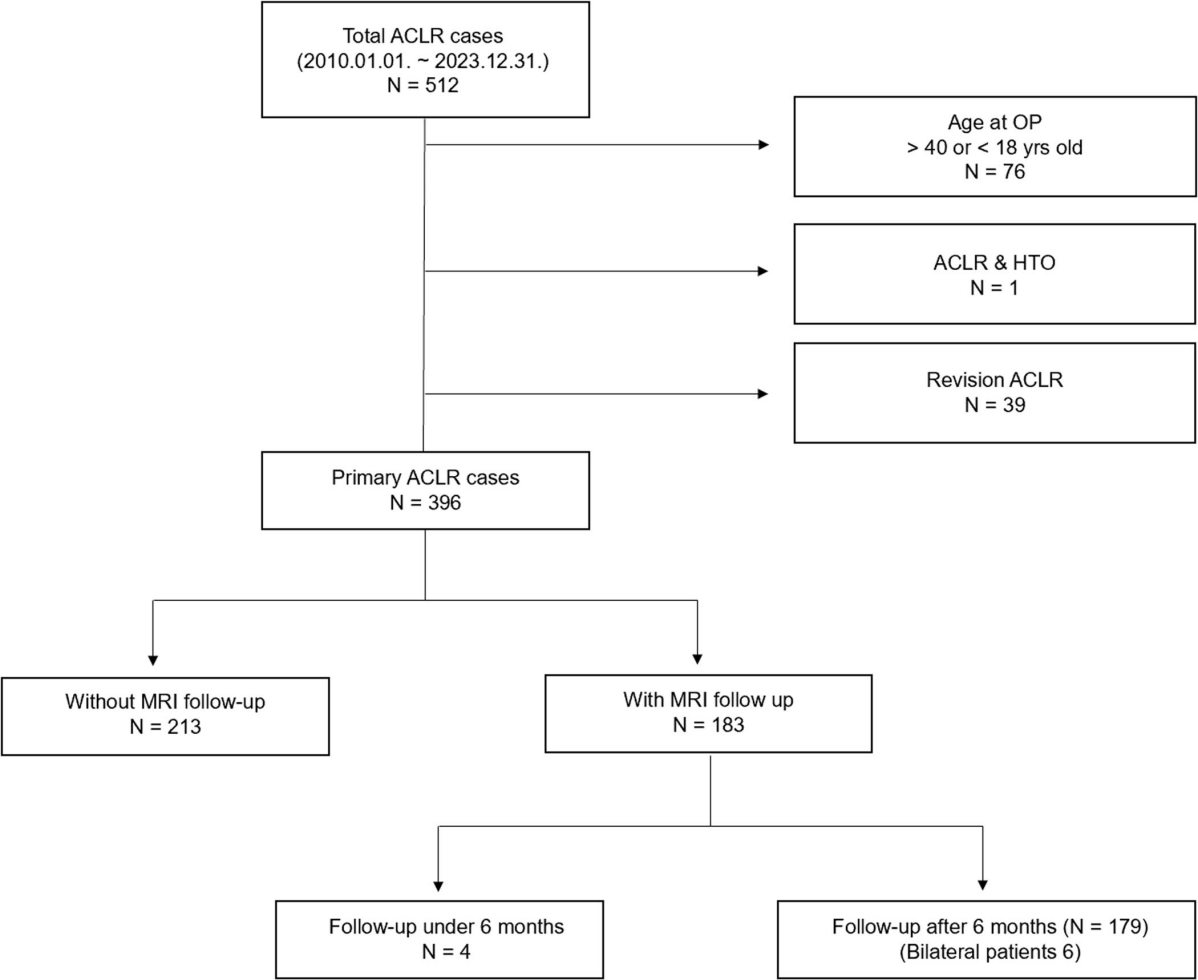
Flowchart of cohort selection for ACLR cases with MRI follow‐up. ACLR, anterior cruciate ligament reconstruction; MRI, magnetic resonance imaging.

A total of 396 primary ACLR cases were investigated, of which 179 cases had MRI follow‐up (Figure 4). In both groups, the proportion of males was approximately 80%, and the mean age was around 26.2 and 26.0 years, respectively (Table 1).

**Table 1:** Characteristics of the study population.

Variables	Total primary ACL cases	With MRI cases
	*N* = 396	*N* = 179
Age, years	26.2	26.0
Gender		
Male	329 (83.1)	142 (79.3)
Female	67 (16.9)	37 (20.7)
Location of surgery		
Right	201 (52.2)	98 (54.7)
Left	195 (47.8)	81 (45.3)
Body mass index (kg/m^2^)	24.3 (22.4–26.9)	24.1 (22.3–26.8)
Interval		
Trauma to operation (days)	29.0 (12.0–117.5)	27.0 (9.0–112.5)
MRI follow‐up (months)	‐	23.4 (13.2–41.5)

*Note*: Values were presented *n* (%) or mean or median (interquartile range).

Abbreviation: ACL, anterior cruciate ligament.

**Figure 5:**
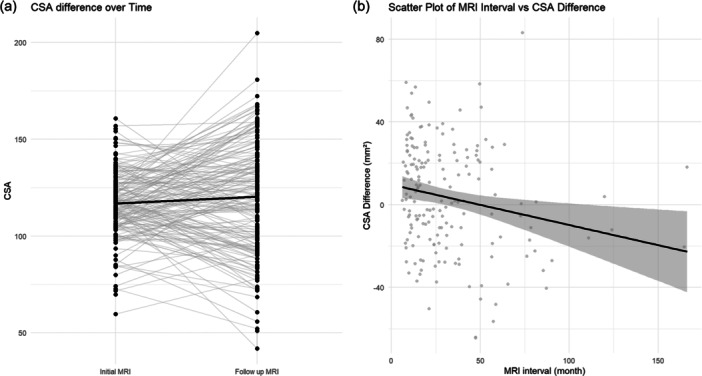
(a) CSA difference over time. The *x*‐axis represents the time points of MRI scans, and the *y*‐axis represents the CSA values. The grey lines show the individual trajectories for each patient, with each black point corresponding to a specific measurement at that time point. The black line indicates the mean CSA value across all patients at each time point. (b) Scatter plot of MRI interval and CSA difference. The *x*‐axis represents the MRI interval in months, while the *y*‐axis represents the CSA difference in mm^2^. The grey points represent individual data points. The black line is the fitted linear regression line, illustrating the trend between the MRI interval and CSA difference. The shaded grey area around the red regression line represents the standard error of the estimate, indicating the uncertainty in the regression model. CSA, cross‐sectional area; MRI, magnetic resonance imaging.

The CSA measured on the follow‐up MRI showed an increase of approximately 3.49 mm^2^ (95% CI: −0.40 to 7.37, *p* value 0.08) compared to the initial MRI, although this change was not statistically significant. Interestingly, CSA actually decreased in 81 cases, representing approximately 45.2% of the cases (Table 2, Figure 5a). When linear regression analysis was performed, adjusting for the interval between MRI scans, it was concluded that for every 1‐month increase in the interval, the difference in CSA decreased by 0.195 mm^2^ (95% CI: −0.337 to −0.053, *p* value 0.007) (Table 3, Figure 5b). Furthermore, this result remained statistically significant even after adjusting for age, gender, BMI and trauma interval.

**Table 2:** Mean width and CSA at both MRI scans.

	Initial MRI	Follow‐up MRI	Difference	*p* **value**
Coronal view (mm)	10.57	10.52	−0.05	
Sagittal view (mm)	11.02	11.33	0.31	
CSA (mm^2^)	116.76	120.25	3.49	0.08
No increase in area (N)	81			

Abbreviations: CSA, cross‐sectional area; MRI, magnetic resonance imaging.

**Table 3:** Linear regression of CSA difference with crude and adjusted values for various variables.

	Crude (95% CI)	Adjusted (95% CI)
Age (years)	−0.047 (−0.706 to 0.611)	−0.090 (−0.747 to 0.567)
Gender		
Female	Reference	Reference
Male	8.298 (−1.245 to 17.841)	8.328 (−1.255 to 17.911)
BMI (kg/m^2^)	0.074 (−0.779 to 0.926)	0.107 (−0.742 to 0.956)
Time interval		
Trauma to operation (days)	0.000 (−0.007 to 0.007)	0.000 (−0.007 to 0.007)
MRI follow‐up (months)*	**−0.195** * **(−0.337 to −0.053)**	**−0.196** * **(−0.39 to −0.053)**

*Note*: For age, BMI and time interval, the change in CSA for each unit increase is described. For gender, female is used as the reference, and the difference in CSA between male and female is noted.

Abbreviations: BMI, body mass index; CI, confidence interval; CSA, cross‐sectional area; MRI, magnetic resonance imaging.

* Significant values.

**Figure 6:**
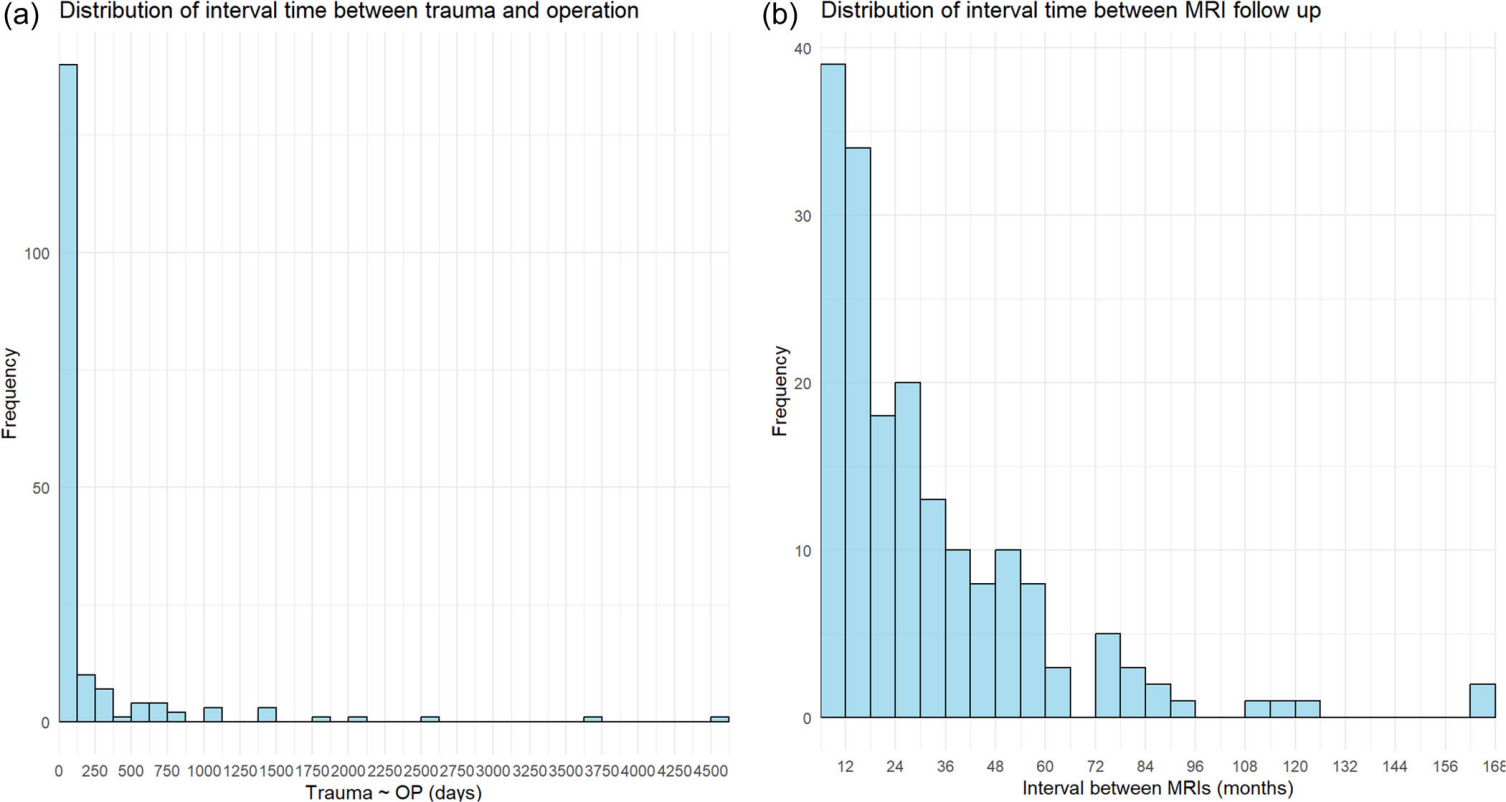
Distribution of interval times: (a) between trauma and operation (in days), and (b) between MRI follow‐ups (in months). MRI, magnetic resonance imaging.

The period from trauma to surgery varied, ranging from a case where surgery was performed the day after the trauma to a case where the patient received conservative treatment for over 10 years. However, 71.5% of the patients underwent surgery within 90 days (Figure 6a), and it was confirmed that tunnel widening was not significantly affected by the trauma‐to‐surgery interval (Table 3).

The mean interval between the post‐surgery MRI and the follow‐up MRI was 21 months, with the longest follow‐up MRI taken after approximately 166 months (13–14 years) (Table 1, Figure 6b). Four patients had follow‐up MRIs taken within 6 months (Figure 4). One patient was suspected of having an infection and underwent imaging at 3 months, while another had imaging at 4 months after experiencing pain post‐exercise, although the ACL was found to be intact. The remaining two patients began exercising 2–3 months after surgery, resulting in re‐rupture of the graft, and ultimately required revision surgery.

Complications were analyzed based on the follow‐up MRIs and additional surgeries performed during the outpatient follow‐up period after ACLR. Out of 396 patients, graft failure was identified on follow‐up MRI in 21 patients, approximately 5.3%. Among them, 17 patients underwent revision surgery in this institution, while 4 patients were lost to follow‐up. In addition to the patients who had meniscus tears at the time of ACLR, two patients were found to have new meniscus tears on follow‐up MRI and subsequently underwent surgery. One had a medial meniscus bucket handle tear at 4 years post‐surgery and underwent repair, while the other underwent partial lateral meniscectomy. The number of patients with cartilage injuries and Cyclops lesions was 6 each (Table 4).

**Table 4:** Complications in total primary ACL cases.

Complication	Number of cases (%)
Graft failure	21 (5.3%)
Meniscus injury	2 (0.5%)
Cartilage injury	6 (1.5%)
Cyclops	6 (1.5%)
Infection	2 (0.5%)

Abbreviation: ACL, anterior cruciate ligament.

## DISCUSSION

The CSA increased by approximately 3.49 mm^2^ on follow‐up MRI, although this change was not statistically significant (Table 2, Figure 5a). After adjusting for the follow‐up period until MRI imaging, it was observed that the difference in CSA gradually decreased (Table 3, Figure 5b). Graft failure occurred in approximately 5.3% of cases, and complications requiring additional surgery were observed in less than 2% of cases (Table 4).

In a prospective cohort of 18 ACLR patients, tibial tunnel width was assessed using MRI. Measurements taken approximately 0.5 cm below the joint‐tunnel interface revealed wider dimensions than at the distal portion, with gradual narrowing observed after 24 months post‐surgery [29]. Recent studies have reported that MRI analyses of patients who underwent ACLR using quadriceps autograft or bone‐patellar tendon‐bone (BTB) autograft showed no significant femoral and tibial tunnel widening approximately 1 year post‐surgery. Notably, in BTB cases, the tibial tunnel width even decreased, which was interpreted as a potential advantage due to bony ingrowth [2]. In contrast, in patients who underwent ACLR with Tibialis anterior tendon allograft fixed using a suspensory device and bioabsorbable screws, serial plain radiographs indicated that the femoral tunnel width increased by approximately 3 mm up to 2 years post‐surgery [14]. Previous studies have also supported the potential impact of bony ingrowth, noting that tunnel widening was reduced when autogenous bone plugs were used compared to bioabsorbable interference screws [11]. In this study, the tibial tunnel aperture area was 120 mm^2^, smaller than the 168.8 mm^2^ reported in the prior study [16], and despite using allogenic bone plugs, tibial tunnel widening was absent in 45.3% of cases. Taken together, these findings suggest that the use of Achilles tendon allograft and calcaneus bone plugs in this study may have contributed to the prevention of tibial tunnel widening.

**Figure 7:**
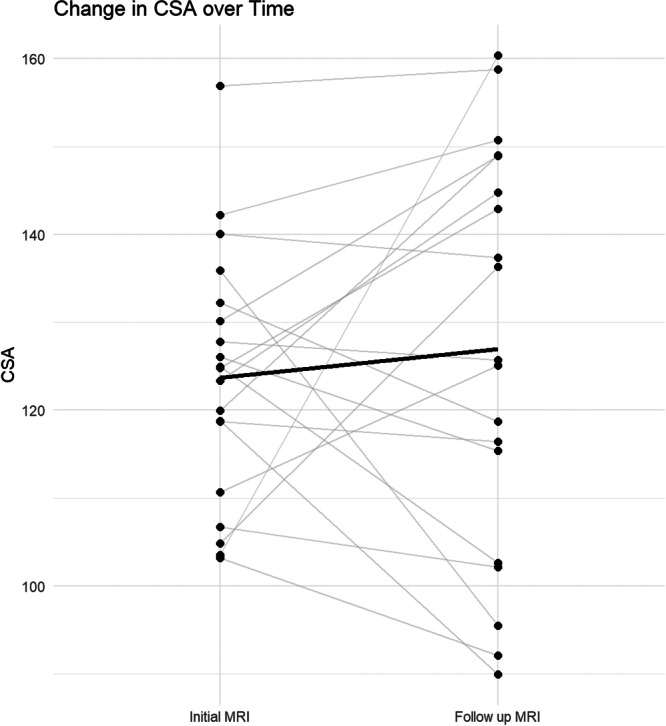
CSA difference over time in patients with failed grafts. The *x*‐axis represents the time points of MRI scans, while the *y*‐axis represents the CSA values. The grey lines show the individual trajectories for each patient, with each black point corresponding to a specific measurement at that time point. The black line indicates the mean CSA value across all patients at each time point. CSA, cross‐sectional area; MRI, magnetic resonance imaging.

Hamstring autografts used in ACLR procedures are associated with rotational instability and low‐grade infection, which can increase the risk of re‐rupture [3, 8, 13, 19, 32]. To address this issue, this study utilized allografts to improve stability and reduce the risks of tunnel widening and graft failure. However, despite these efforts, the re‐rupture rate was approximately 5.3% (21 out of 396 cases), which is similar to the graft failure rate observed in studies using autografts with the traditional transtibial technique [4]. As a result, a subgroup analysis was conducted on the 21 patients who experienced re‐rupture to investigate the causal relationship between graft re‐rupture and tunnel widening. Two of these patients were re‐ruptured only four months after surgery due to soccer and trauma‐related activities. The remaining 19 patients experienced graft failure after an average of 37.4 months. Among them, five were military personnel and four were professional athletes, including two judokas, a soccer player and a wrestler. Of the remaining 12 patients, 9 experienced re‐rupture while participating in high‐contact sports, specifically soccer or judo. The average age of these 19 patients was 22.6 years, with an average BMI of 26.4 kg/m^2^. On follow‐up MRI, their CSA increased by 3.25 mm^2^, but this change was not statistically significant (95% CI: −7.94 to 14.44, *p* value 0.55) (Table 5, Figure 7). No significant results were obtained from linear regression analyses adjusted for multiple variables. Therefore, the re‐rupture in this study is considered to be more likely a consequence of excessive activities by patients engaging in high‐intensity exercise, rather than being caused by tunnel widening.

**Table 5:** Mean width and CSA at both MRI scans in patients with failed grafts.

	Initial MRI	Follow‐up MRI	Difference	*p* **value**
Coronal view (mm)	10.77	11.17	0.4	
Sagittal view (mm)	11.47	11.32	−0.15	
CSA (mm^2^)	123.70	126.98	3.25	0.55
No increase in area (N)	10			

Abbreviations: CSA, cross‐sectional area; MRI, magnetic resonance imaging.

Complication rates for meniscus and cartilage damage post‐ACLR were 0.5% and 1.5%, respectively—lower than the rates of 11.9% and 6.7% for similar complications reported in larger cohorts [24]. This is likely due to the fat pad preservation technique used in this procedure, which contributes to the protection of the cartilage and meniscus.

The incidence of Cyclops lesions following ACLR is reported to range from 2% to 11%, with some studies reporting up to 20% during second‐look arthroscopy [9, 18]. In this cohort, Cyclops was identified in six cases, all of which were successfully treated via surgical excision. These findings not only demonstrate the efficacy of the surgical approach employed but also further reinforce the conclusions of previous research, which suggested that this method helps prevent the formation of Cyclops [23].

Infection rates associated with ACLR are approximately 1%, with higher risks linked to hamstring autografts due to longer surgical times and site haematomas [32]. Additionally, about 10%–16% of harvested grafts were found to be bacterial contaminated, and approximately 2.4% of them led to post‐operative infections [20]. In our study, two patients experienced a suspected infection. The first case involved a patient who underwent irrigation, screw replacement, and antibiotic therapy, with symptoms resolving within 4 months. However, this patient had an MRI at 3 months post‐operation and was subsequently excluded from the final study. The second patient, an athlete, presented approximately 6 years post‐surgery with persistent effusion and swelling during exercise. This patient underwent irrigation of the area where the tibia screw was removed, along with a tibial tunnel allograft bone graft, resulting in symptom improvement.

This study acknowledges several limitations. The MRI follow‐up intervals showed considerable variability, ranging from 6 months to approximately 13 years post‐surgery. Additionally, about 213 cases were excluded due to the absence of follow‐up MRIs during outpatient visits. Despite this limitation, the inclusion of 179 cases from a single institution represents a relatively large sample size, which helps address this issue effectively [11, 22, 29]. Second, trauma dates were recorded based on patients' memory, introducing the possibility of recall bias. This issue is particularly relevant for patients who underwent prolonged conservative treatment before surgery. However, 71.5% of cases (128 out of 179) involved surgery within 90 days of injury (Figure 6a), suggesting that inaccuracies in trauma dates are unlikely to significantly influence the study's conclusions. Another limitation is the absence of follow‐up stress X‐rays for all patients at the time of MRI, leading to missing side‐to‐side difference (STSD) data, which were excluded from the analysis. Similarly, patient‐reported knee scores were not included in this study. Nevertheless, pain and instability were consistently assessed during outpatient visits, and detailed evaluations, including MRIs, were performed for patients with symptoms. If abnormalities were detected on MRI, arthroscopic examination was performed, and surgery was conducted if necessary. These findings were incorporated into the complication analysis of this study. Thus, the absence of these specific datasets is unlikely to alter the overall findings of this study. Future research will aim to include these parameters to further validate the exceptional long‐term outcomes of this surgical technique.

## CONCLUSION

The CSA was measured to be larger on follow‐up MRI, but this increase was not statistically significant, and there was a tendency for the difference to gradually decrease over time. The complication rate associated with the technique used in this study was similar to or lower than that reported in previous studies, further supporting the superiority of this surgical approach.

## AUTHOR CONTRIBUTIONS

All authors contributed to the study conception and design. Material preparation, data collection and analysis were performed by Siyeon Kim, Dae Keun Suh and Dong won Suh. The first draft of the manuscript was written by Siyeon Kim and Dae Keun Suh. All authors commented on previous versions of the manuscript and approved the final manuscript.

## CONFLICT OF INTEREST STATEMENT

The authors declare no conflicts of interest.

## ETHICS STATEMENT

This study was performed in line with the principles of the Declaration of Helsinki. The Institutional Review Board approved this study (P01‐202411‐01‐049) and waived the requirement for informed consent because we used de‐identified data routinely collected during clinic visits. Patients signed informed consent regarding publishing their data and photographs.

## Data Availability

The data sets generated and analyzed during the current study are available from the corresponding author on reasonable request.
